# Secondary osteoporosis in a young woman: the hidden impact of functional hypothalamic amenorrhea

**DOI:** 10.12701/jyms.2026.43.23

**Published:** 2026-03-18

**Authors:** Hyunji Reem, Hyebin Kim, Seung Min Chung

**Affiliations:** 1 Yeungnam University College of Medicine, Daegu, Korea; 2 Department of Internal Medicine, Yeungnam University Medical Center, Daegu, Korea; 3 Division of Endocrinology and Metabolism, Department of Internal Medicine, Yeungnam University College of Medicine, Daegu, Korea

## Case presentation

**Fig. 1.**
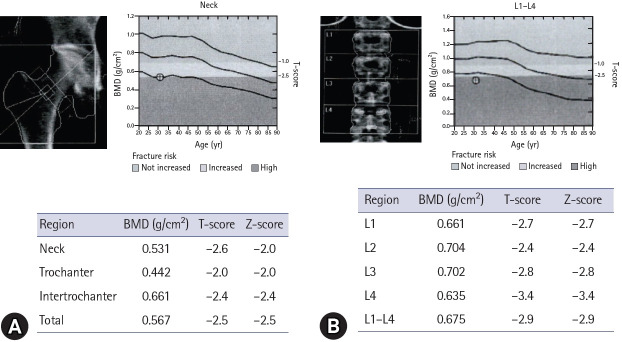
Dual-energy X-ray absorptiometry scan showing low BMD before treatment. (A) Femoral neck scan shows a T-score of −2.6 and a Z-score of −2.0. (B) Lumbar spine scan (L1–L4) shows a T-score and Z-score of −2.9. In this 31-year-old patient who is premenopausal with functional hypothalamic amenorrhea, these findings, particularly a T-score of ≤−2.5 in a young adult with a chronic condition causing metabolic bone alterations, are diagnostic of secondary osteoporosis. This highlights that her skeletal health was severely compromised due to hypoestrogenism and chronic energy deficiency caused by inadequate nutritional intake. BMD, bone mineral density.

A 31-year-old woman visited the Department of Endocrinology and Metabolism at our university hospital complaining of pain and numbness in her wrists, ankles, knees, and lower legs. A dual-energy X-ray absorptiometry (DXA) scan at a local hospital showed lower bone mineral density (BMD) than expected for the patient’s age, with Z-scores of −2.0 for the femoral neck and −2.9 for the lumbar spine (Fig. 1). The patient sought an explanation for early-onset bone loss.

The patient was 153 cm tall, weighed 45 kg, and had a body mass index (BMI) of 19.2 kg/m². She did not smoke but consumed three drinks of alcohol per week. The patient experienced menarche at 13 years of age and amenorrhea at 19 years of age, when she reported an increased body weight (60 kg) and started a restrictive diet. Subsequently, following a period of severe anorexia, she developed episodes of binge eating followed by self-induced vomiting, resulting in a total weight loss of up to 36 kg. At 23 years old, transvaginal ultrasonography revealed no structural uterine abnormalities. The patient is single and has no history of pregnancy or lactation. She reported taking statins because of high cholesterol levels. Additionally, she did not report gastrointestinal symptoms, a history of kidney stones or surgeries, or a family history of osteoporosis or fractures. The laboratory examination results are presented in Table 1.

## Differential diagnosis

Since its introduction, DXA has revolutionized the diagnosis, management, and monitoring of osteoporosis, serving as a pivotal tool for assessing BMD in clinical practice [1]. The International Society for Clinical Densitometry recommends using Z-scores for children, adolescents, women who are premenopausal, and men aged <50 years [1]. In these populations, a Z-score of ≤−2.0 is defined as “BMD below the expected range for age” [1]. However, to maintain consistency with the World Health Organization operational definition, a T-score of ≤−2.5 at the spine or hip is considered diagnostic of osteoporosis in young adults with chronic conditions that cause metabolic bone alterations [2]. In the present case, the patient’s T-scores were −2.6 at the femoral neck and −2.9 at the lumbar spine, confirming the diagnosis of osteoporosis (Fig. 1). The following diagnoses were considered to identify the underlying etiology of this secondary osteoporosis:

## 1. Primary hyperparathyroidism

Patients with primary hyperparathyroidism typically seek medical attention because of low BMD accompanied by inappropriately elevated parathyroid hormone (PTH) levels, despite the presence of hypercalcemia [3]. The patient’s serum intact-PTH level was 27.5 pg/mL (range, 15.0–65.0 pg/mL), total calcium level was 9.0 mg/dL (range, 8.6–10.6 pg/mL), and 25-hydroxyvitamin D level was 32.4 ng/mL (range, >30.0 ng/mL). Her serum creatinine level was normal (0.58 mg/dL), and she had no medical history of kidney stones. Therefore, hyperparathyroidism was less likely to have caused osteoporosis in this case.

## 2. Hyperthyroidism

Thyroid hormones stimulate bone resorption, which results in increased cortical bone porosity and reduced trabecular bone volume. The net effect is osteoporosis and increased fracture risk in patients with overt hyperthyroidism [4]. The present patient was euthyroid (thyroid-stimulating hormone level, 0.92 mU/L; free thyroxine level, 13.55 pmol/L; and total triiodothyronine level, 1.28 nmol/L). Therefore, hyperthyroidism was unlikely to be the cause of osteoporosis in this case.

## 3. Cushing syndrome

Cushing syndrome, which can be adrenocorticotropic hormone (ACTH)-dependent (e.g., pituitary adenoma or ectopic ACTH secretion by nonpituitary tumors) or ACTH-independent (e.g., adrenal adenoma, carcinoma, or nodular hyperplasia), results in chronic hypercortisolemia and various endocrine disorders (e.g., central obesity, impaired glucose tolerance, hypertension, and osteoporosis) [5]. In patients with symptoms suggestive of hypercortisolism, a 24-hour urinary free cortisol test, an overnight dexamethasone suppression test, or a midnight salivary cortisol test should be conducted. The patient had no signs or symptoms suggestive of hypercortisolism, such as moon face, buffalo hump, or easy bruising. The patient denied a history of glucocorticoid use. Therefore, in this case, Cushing syndrome was less likely to cause osteoporosis.

## 4. Functional hypothalamic amenorrhea

The patient had amenorrhea for 12 years, with a persistent negative energy balance due to anorexia nervosa. Women affected by functional hypothalamic amenorrhea (FHA) may experience significant bone loss and issues with the restoration of healthy bone density [6]. During adolescence, this condition mainly reduces bone formation; however, it also increases bone resorption in adults, leading to impaired BMD, structure, and microarchitecture [7]. The patient’s serum follicle-stimulating hormone level was 5.73 IU/L (range, 3.40–12.00 IU/L), estradiol level was 70.19 pg/mL (range, 40.00–500.00 pg/mL) during the follicular phase, and prolactin level was 3.62 ng/mL (range, 3.70–17.20 ng/mL). Given her medical history and laboratory results, it was highly likely that her osteoporosis was caused by FHA.

## Diagnosis

Based on the differential diagnoses discussed above, the patient was diagnosed with secondary osteoporosis attributable to overlapping FHA and anorexia nervosa, and all other conditions were excluded.

## Treatment and prognosis

The patient underwent multidisciplinary management involving an endocrinologist, gynecologist, and psychologist. She participated in regular consultations to restore her energy balance and alleviate psychological stress. Treatment included oral contraceptives (low-dose estrogen/progesterone), calcium and vitamin D supplementation, and antiresorptive therapy with denosumab to enhance BMD.

**Fig. 2.**
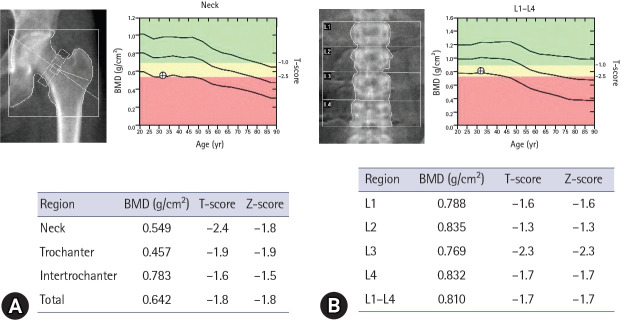
Dual-energy X-ray absorptiometry scan showing improved BMD after 1 year of treatment. (A) Femoral neck scan shows an improved T-score of −2.4 and a Z-score of −1.8. (B) Lumbar spine scan shows a T-score and Z-score of −1.7. This progress reflects the successful restoration of energy balance and the efficacy of sequential antiresorptive therapy. The recovery at both sites illustrates the potential for skeletal restoration when the underlying etiology is appropriately addressed alongside pharmacological intervention. BMD, bone mineral density.

After 1 year, the patient achieved a 9-kg weight gain (height, 154 cm; weight, 53.7 kg; BMI, 22.6 kg/m²), and spontaneous menstruation resumed without further need for oral contraceptives. Z-scores for the femoral neck and lumbar spine improved to −1.8 and −1.7, respectively (Fig. 2). Oral contraceptives were discontinued and denosumab was replaced with zoledronate, an intravenous bisphosphonate. The patient has received continuous follow-up, and no significant complications have been reported to date.

## Discussion

This case shows that secondary osteoporosis can occur in young women because of FHA, even without other endocrine disorders.

In young adults, osteoporosis is usually caused by reversible conditions, such as endocrine abnormalities (e.g., hyperparathyroidism, thyrotoxicosis, hypercortisolism, hypogonadism, and pituitary disorders), which can significantly lower bone density and increase fracture risk. When low-trauma fractures or unexpected low BMD is detected, a thorough workup with history, physical examination, and targeted laboratory tests—including calcium, phosphate, and various hormones such as PTH, thyroid function tests, morning cortisol, gonadotropins, sex steroids, prolactin, and insulin-like growth factor-1 (IGF-1)—is crucial [2].

FHA is a common cause of secondary osteoporosis in young women with chronic energy deficiency and low estrogen levels. FHA is the suppression of the hypothalamic-pituitary-ovarian axis in response to low energy consumption, excessive exercise, or psychological stress, resulting in low estradiol levels and amenorrhea, while concomitant reductions in IGF-1 and alterations in leptin and cortisol further impair bone formation and microarchitecture [8]. Management of FHA-related bone loss must prioritize the restoration of energy balance and menstrual function through nutritional rehabilitation, weight gain, reduction in excessive physical activity, and psychological support. In such cases, hormone replacement is considered as an adjunct rather than a substitute for lifestyle correction. Physiologic transdermal 17β-estradiol with cyclic oral progesterone is more favorable for bone density than standard combined oral contraceptives, whereas potent antiresorptive or anabolic agents should be reserved for women with very low BMD or recurrent fractures who remain hypogonadal despite optimal lifestyle and hormonal interventions, given concerns regarding pregnancy and long-term skeletal retention of the drugs [9]. Because denosumab provides rapid BMD gain but lacks skeletal retention, discontinuation can trigger a rebound increase in bone resorption. Sequential therapy with bisphosphonates is essential to prevent this because their long skeletal half-life ensures BMD stability through continued osteoclast suppression [10].

In conclusion, this case highlights the importance of early recognition and comprehensive management of secondary osteoporosis in young women, particularly when it is associated with FHA and chronic energy deficiency. A multidisciplinary approach tailored to address both the underlying endocrine and psychological factors, along with targeted pharmacological therapy, can substantially improve bone health and overall prognosis. Continued monitoring and individualized care are essential to prevent recurrence and ensure long-term skeletal and reproductive health in this population.

## Educational pearls

### 1. Comprehensive screening for secondary causes is mandatory in young women with low bone mineral density

Unlike postmenopausal osteoporosis, low BMD in young women is frequently caused by secondary potentially reversible endocrine disorders. Clinicians must perform a thorough workup to evaluate conditions such as hyperparathyroidism, hyperthyroidism, Cushing syndrome, and FHA.

### 2. Functional hypothalamic amenorrhea is a major cause of secondary osteoporosis in young women

Chronic energy deficiency, as observed in anorexia nervosa, suppresses the hypothalamic-pituitary-ovarian axis. This causes profound hypoestrogenism and metabolic disturbances, which significantly increase bone resorption and impair bone formation, resulting in severely low BMD.

### 3. A multidisciplinary approach focused on restoring energy balance is the cornerstone of management

Pharmacological interventions should be considered adjunctive to nutritional rehabilitation. A coordinated multidisciplinary approach involving endocrinologists, gynecologists, and psychologists is essential to address the underlying energy deficits, restore menstrual function, and manage psychopathology, which are prerequisites for skeletal recovery.

### 4. Sequential therapy is essential to mitigate the rebound increase in bone resorption after denosumab discontinuation

As denosumab lacks skeletal retention, its inhibitory effect on osteoclasts is rapidly reversed upon cessation, triggering a rebound phenomenon characterized by rapid bone loss. Transitioning to a bisphosphonate is required to maintain BMD stability through the continued suppression of osteoclast activity.

## Question 1

A 31-year-old woman presents with pain and numbness in the wrists, ankles, knees, and lower legs. Dual-energy X-ray absorptiometry reveals a lumbar spine Z-score of –2.9. The patient had a 12-year history of amenorrhea related to anorexia nervosa. Which of the following is the most likely pathophysiological mechanism underlying the patient’s low bone mineral density?

- A. Excessive secretion of parathyroid hormone
- B. Increased thyroid hormones
- C. Suppression of the hypothalamic-pituitary-ovarian axis
- D. Reduced intestinal calcium absorption
- E. Excessive secretion of cortisol

C

## Question 2

After 1 year of antiresorptive therapy with denosumab, the patient was switched to zoledronate. What is the primary clinical rationale for this sequential therapy?

- A. To prevent the rebound increase in bone resorption associated with denosumab discontinuation
- B. To further stimulate osteoblast activity and bone formation
- C. To maximize the suppression of osteoclast-mediated bone loss
- D. To allow for the resumption of spontaneous menstruation
- E. To avoid the development of neutralizing antibodies against denosumab

A

## Figures and Tables

**Table 1.** Baseline laboratory findings

Parameter	Result	Reference range
Body mass index (kg/m^2^)	19.2	18.5–23.0
Albumin (g/dL)	4.65	3.50–5.00
Creatinine (mg/dL)	0.58	0.50–0.90
Total calcium (mg/dL)	9.0	8.6–10.6
Inorganic phosphorus (mg/dL)	4.1	2.5–4.5
25(OH)D (ng/mL)	32.4	>30.0
Intact PTH (pg/mL)	27.5	15.0–65.0
CTX (ng/mL)	0.169	0.025–0.537
P1NP (ng/mL)	26.20	15.13–73.87
TSH (mIU/L)	0.92	0.30–4.00
Free thyroxine (pmol/L)	13.55	10.00–25.00
Total triiodothyronine (nmol/L)	1.28	1.20–2.80
Estradiol (pg/mL)	70.19	Follicular phase: 40.00–500.00
FSH (IU/L)	5.73	Follicular phase: 3.40–12.00
Prolactin (ng/mL)	3.62	3.70–17.20

25(OH)D, 25-hydroxyvitamin D; PTH, parathyroid hormone; CTX, C-terminal telopeptide of type 1 collagen; P1NP, procollagen type 1 N-terminal propeptide; TSH, thyroid-stimulating hormone; FSH, follicle-stimulating hormone.
